# Enhancing Safety in Epilepsy Surgery (EASINESS): Study Protocol for a Retrospective, Multicenter, Open Registry

**DOI:** 10.3389/fneur.2021.782666

**Published:** 2021-12-13

**Authors:** Richard Drexler, Sharona Ben-Haim, Christian G. Bien, Valeri Borger, Francesco Cardinale, Alexandre Carpentier, Fernando Cendes, Sarat Chandra, Hans Clusmann, Albert Colon, Marco de Curtis, Daniel Delev, Giuseppe Didato, Lasse Dührsen, Jibril Osman Farah, Marc Guenot, Saadi Ghatan, Claire Haegelen, Hajo Hamer, Jason S. Hauptmann, Rosalind L. Jeffree, Thilo Kalbhenn, Josua Kegele, Niklaus Krayenbühl, Johannes Lang, Bertrand Mathon, Georgios Naros, Julia Onken, Fedor Panov, Christian Raftopoulos, Franz L. Ricklefs, Kim Rijkers, Michele Rizzi, Karl Rössler, Olaf Schijns, Ulf C. Schneider, Andrea Spyrantis, Adam Strzelczyk, Stefan Stodieck, Manjari Tripathi, Sumeet Vadera, Mario A. Alonso-Vanegas, José Géraldo Ribero Vaz, Jörg Wellmer, Tim Wehner, Manfred Westphal, Thomas Sauvigny

**Affiliations:** ^1^Department of Neurosurgery, University Medical Center Hamburg-Eppendorf, Hamburg, Germany; ^2^Department of Neurosurgery, University of California San Diego, San Diego, CA, United States; ^3^Epilepsy Centre Bethel, Krankenhaus Mara, Bielefeld, Germany; ^4^Department of Neurosurgery, University Hospital Bonn, Bonn, Germany; ^5^“C. Munari” Epilepsy Surgery Center, Niguarda Hospital, Milan, Italy; ^6^Department of Neurosurgery, Pitié-Salpêtrière Hospital, Paris, France; ^7^Department of Neurology, University of Campinas, Campinas, Brazil; ^8^Department of Neurosurgery, AIIMS, New Delhi, India; ^9^Department of Neurosurgery, University Hospital RWTH Aachen, Aachen, Germany; ^10^School for Mental Health and Neuroscience (MHeNS), University Maastricht (UM), Maastricht, Netherlands; ^11^Epilepsy Unit, IRCCS “C. Besta” Neurological Institute Foundation, Milan, Italy; ^12^The Walton Centre NHS Foundation Trust, Liverpool, United Kingdom; ^13^Department of Functional Neurosurgery, P. Wertheimer Hospital, Hospices Civils de Lyon, Lyon, France; ^14^Department for Neurosurgery, University of Lyon, Lyon, France; ^15^Department of Neurosurgery, Icahn School of Medicine at Mount Sinai, New York, NY, United States; ^16^Epilepsy Center, Department of Neurology, University of Erlangen-Nürnberg, Erlangen, Germany; ^17^Department of Neurosurgery, University of Washington, Seattle, WA, United States; ^18^Department of Neurosurgery, Royal Brisbane and Womens Hospital, Brisbane, QLD, Australia; ^19^Herston Clinical School, University of Queensland, Brisbane, QLD, Australia; ^20^Department of Neurosurgery (Evangelisches Klinikum Bethel), Bielefeld University, Medical School, Bielefeld, Germany; ^21^Department of Neurology and Epileptology, Hertie Institute of Clinical Brain Research, University of Tubingen, Tubingen, Germany; ^22^Division of Pediatric Neurosurgery, University Children's Hospital Zurich, University of Zurich, Zurich, Switzerland; ^23^Department of Neurosurgery, Universitätsmedizin Charité-Berlin, Berlin, Germany; ^24^Department of Neurosurgery, University Hospital St-Luc, Université Catholique de Louvain (UCL), Brussels, Belgium; ^25^Department of Neurosurgery, Academic Center for Epileptology UMC Maastricht, Maastricht, Netherlands; ^26^Department of Neurosurgery, Fondazione IRCCS Istituto Neurologico Carlo Besta, Milan, Italy; ^27^Department of Neurosurgery, Medical University Vienna, Vienna, Austria; ^28^School for Mental Health and Neuroscience (MHeNS), University Maastricht (UM), Maastricht, Netherlands; ^29^Department of Neurosurgery and Epilepsy Center Frankfurt Rhine-Main, Center of Neurology and Neurosurgery, Goethe-University Frankfurt, Frankfurt, Germany; ^30^Hamburg Epilepsy Center, Protestant Hospital Alsterdorf, Department of Neurology and Epileptology, Hamburg, Germany; ^31^Department of Neurological Surgery, University of California Irvine, Irvine, CA, United States; ^32^National Institute of Neurology and Neurosurgery, Manuel Velasco Suarez, Mexico City, Mexico; ^33^Ruhr - Epileptology, Department of Neurology, University Hospital Knappschafts-Krankenhaus, Ruhr - University Bochum, Bochum, Germany

**Keywords:** epilepsy, epilepsy surgery, temporal lobe epilepsy, outcome, benchmark, seizure outcome, amygdalohippocampectomy, anteromedial resection

## Abstract

**Introduction:** Optimizing patient safety and quality improvement is increasingly important in surgery. Benchmarks and clinical quality registries are being developed to assess the best achievable results for several surgical procedures and reduce unwarranted variation between different centers. However, there is no clinical database from international centers for establishing standardized reference values of patients undergoing surgery for mesial temporal lobe epilepsy.

**Design:** The Enhancing Safety in Epilepsy Surgery (EASINESS) study is a retrospectively conducted, multicenter, open registry. All patients undergoing mesial temporal lobe epilepsy surgery in participating centers between January 2015 and December 2019 are included in this study. The patient characteristics, preoperative diagnostic tools, surgical data, postoperative complications, and long-term seizure outcomes are recorded.

**Outcomes:** The collected data will be used for establishing standardized reference values (“benchmarks”) for this type of surgical procedure. The primary endpoints include seizure outcomes according to the International League Against Epilepsy (ILAE) classification and defined postoperative complications.

**Discussion:** The EASINESS will define robust and standardized outcome references after amygdalohippocampectomy for temporal lobe epilepsy. After the successful definition of benchmarks from an international cohort of renowned centers, these data will serve as reference values for the evaluation of novel surgical techniques and comparisons among centers for future clinical trials.

**Clinical trial registration:** This study is indexed at clinicaltrials.gov (NT 04952298).

## Introduction

Surgeons strive for the best possible outcome from their operations with the greatest possible chance for recovery and cure of the patients. To optimize the outcome, the continuous monitoring and measurement of key elements of surgical procedures are indispensable. To this end, the number of clinical registries has increased in recent years (1). To evaluate surgical quality, recent initiatives aim to assess the best achievable results for several surgical procedures and reduce unwarranted variation between different centers (2–4). A standardized methodology to describe optimal outcomes in neurosurgery is lacking. The most appealing concept in surgery is a combination of various clinical indicators with risk stratification and a focus on treatment efficacy and adverse events. This offers a more reliable analysis than single-outcome indicators, such as survival or readmission rate (5–10). Nevertheless, an establishment of clinical databases from international centers with an accurate collection of demographic, management, and outcome data for respective surgical procedures is needed. The importance of establishing benchmarks has been demonstrated for various surgical procedures in general and visceral surgery (11–15). As yet, there is very little published about acceptable neurosurgical benchmarks. A benchmark establishes standardized reference values which represent the acceptable common outcome of high-volume centers and can be used for comparison and improvement. Best results are obtained from patients at the lowest risk (10). Therefore, the cohort must be divided into low-risk and high-risk patients according to the defined benchmark criteria that predict a potentially worse postoperative outcome.

The aim of this international multicenter register [Enhancing Safety in Epilepsy Surgery (EASINESS)] is to define robust and standardized outcome references after amygdalohippocampectomy and anteromedial temporal lobectomy for temporal lobe epilepsy. After the successful determination of benchmarks from an international cohort of renowned centers, these data will serve as reference values for the evaluation of novel surgical techniques and comparisons among centers for future clinical trials. They will provide feedback to participating centers to enable deficiencies in care to be addressed, thereby enabling quality improvement. This registry will demonstrate the feasibility and utility of risk-stratified benchmarked data for neurosurgical practice.

## Materials and Methods

### Study Design

The EASINESS is a retrospectively conducted, multicenter, open registry. The study is a procedure-specific registry, initiated by the Department of Neurosurgery, University Medical Center Hamburg-Eppendorf, Germany. The selected members of participating neurosurgical or epilepsy centers will be invited to form a Steering Committee, which will be responsible for the scientific goals and guarantee the reliability of the data analysis. Neurosurgical, neurological, and epilepsy units from all countries are invited to join the trial and to recruit patients if the center- and patient-specific inclusion criteria are met.

### Study Setting and Type of Participants

All patients undergoing mesial temporal lobe epilepsy surgery between January 1, 2015 and December 31, 2019 are included in the study. The following surgical procedures are included: selective amygdalohippocampectomy including anterior parahippocampal gyrus/entorhinal cortex, anteromedial temporal lobe resection including amygdalohippocampectomy with approximately anterior third of the temporal neocortex, amygdala, uncus, hippocampus, anterior parahippocampal gyrus/entorhinal cortex, and anterior temporal lobe resection with two-thirds of the temporal neocortex, amygdala, uncus, hippocampus, and anterior parahippocampal gyrus/entorhinal cortex (16, 17). Patients who went through deep brain stimulation, vagus nerve stimulation, neocortical temporal resection only, or a recurrent resection are not included in this study.

### Center-Specific Inclusion Criteria

The participating centers need to perform 30 or more seizure-specific resections as an average per year during the 5-year study period. This threshold is based on the recommendations of the German Society for Epileptology and the Austrian, German, and Swiss working group on presurgical epilepsy diagnosis and operative epilepsy treatment for certification as an epilepsy center performing epilepsy surgery (18, 19). International centers may be accepted on a case-by-case basis if they meet the alternative local guidelines for epilepsy center definition. The implantation of vagus nerve stimulators was not counted toward the number of seizure-specific procedures (Table 1).

**Table 1:** Inclusion and exclusion criteria for centers and patients to participate in the Enhancing Safety in Epilepsy Surgery (EASINESS) registry.

**Inclusion criteria**	**Exclusion criteria**
Center-specific:	
1. Centers with ≥30 seizure-specific resections (excluding vagus nerve stimulation) as an average per year during the study period	
Patient-specific:	
1. Patients who went through mesial temporal lobe epilepsy surgery, including: a. Selective Amygdalohippocempectomy including anterior parahippocampal gyrus / entorhinal cortex b. Anteromedial temporal lobe resection including amygdalohippocampectomy c. Anterior temporal lobe resection	1. Patients who went through neocortical temporal resection only 2. Patients who went through recurrent resection

### Informed Consent

Written informed consent is not required in this retrospective study since all patient data are processed anonymously. The study was approved by the Institutional Ethics Committee centrally at University Medical Center Hamburg-Eppendorf and local governance approval was obtained by each site as necessary. The patients' data were extracted and analyzed following the declaration of Helsinki.

### Data Collection

According to a standardized template for mesial temporal lobe epilepsy surgery, patient-specific data, surgical details, postoperative complications, and long-term seizure outcome including neuropsychological assessment will be recorded. The data acquisition will be electronic-based (case report form, CRF), and patient-specific data will be de-identified by the study center. The data will then be transferred into an electronic database and further analyses made at the Department of Neurosurgery, University Medical Center Hamburg-Eppendorf.

### Case Report Form

**Figure 1:**
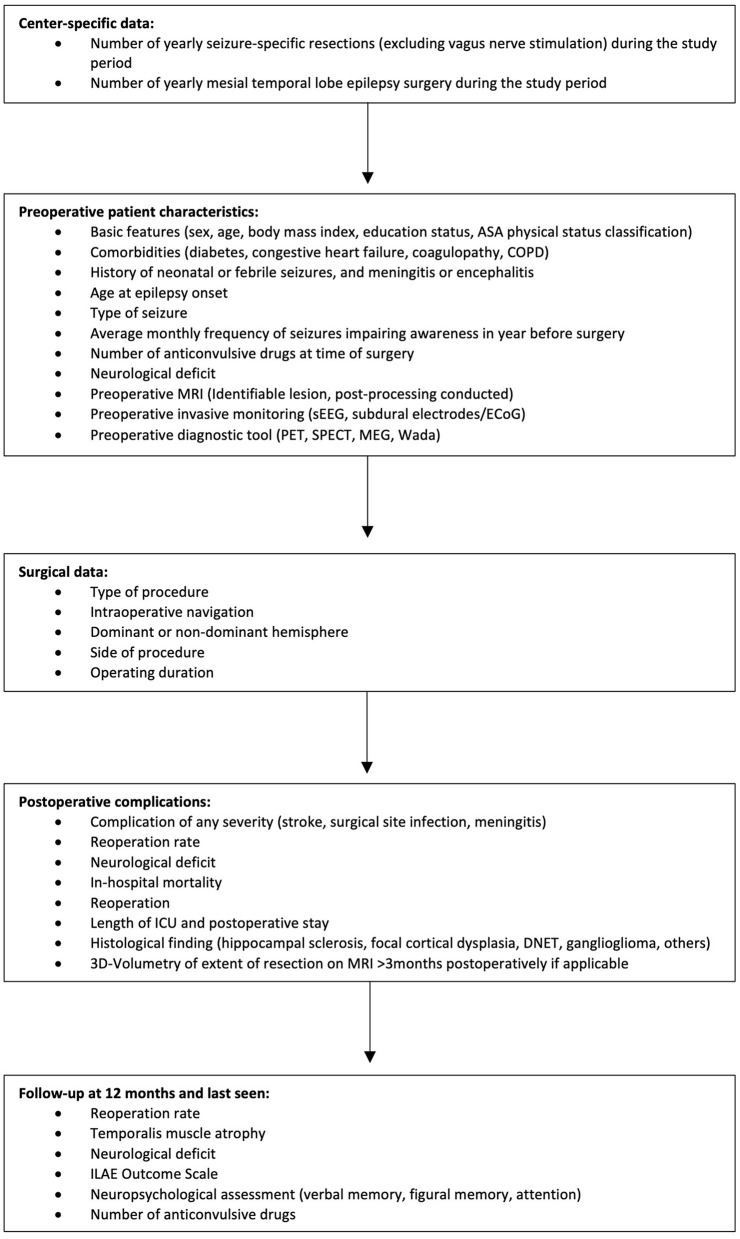
Study protocol of the Enhancing Safety in Epilepsy Surgery (EASINESS) registry.

The specially designed and developed Case Report Form (CRF) consists of four parts (Figure 1).

1. *Patient characteristics:* The first part of the CRF covers preoperative characteristics including patient demographic and baseline characteristics (sex, age, body mass index, educational status, neurological status), comorbidities (diabetes, congestive heart failure, coagulopathy, and COPD), and the American Society of Anesthesiologists (ASA) Physical Status Classification. In addition, seizure-specific features such as a history of neonatal seizure, febrile seizure, encephalitis or meningitis, age at epilepsy onset, type of seizure, average monthly frequency of seizures, and a number of anticonvulsive drugs at the time of surgery are registered. Furthermore, presurgical diagnostic tools (e.g., invasive monitoring, PET, SPECT, MEG) and possible lesions in MRI will be documented.
2. *Surgical data:* This part covers all aspects of mesial temporal lobe epilepsy surgery. Here, type of procedure, intraoperative navigation, resection of the dominant or non-dominant hemisphere, side of resection, and the operating duration are registered.
3. *Postoperative complications:* The fourth part addresses all questions of the outcome until discharge after index surgery. Postoperative complications such as stroke, surgical site infection, meningitis, reoperation, new neurological deficits, and in-hospital mortality will be recorded. In addition, the histological finding of the resected lesion is registered.
4. *Follow-up:* Long-term clinical and seizure outcome after 12 months and an optional later time will be recorded. Here, long-term complications such as reoperation, temporalis muscle atrophy, and neurological deficits are registered. To analyze the seizure-specific outcome after surgery, the International League Against Epilepsy (ILAE) outcome scale, neuropsychological assessment (verbal and figural memory, attention), and the number of antiseizure medications will be documented.

### Data Management

Data collection will be performed locally at each site and de-identified in an independent database, then submitted to the Department of Neurosurgery at University Medical Center Hamburg-Eppendorf (Germany). All results, including patient-specific data, surgical techniques, complications, and long-term outcomes, will be submitted for publication in peer-reviewed journals and/or reported at professional scientific meetings. Every participating center can submit further scientific questions which will be evaluated by the steering committee for approval or rejection. The by-laws of the steering committee are accessible from the principal investigator upon request. This study is indexed at clinicaltrials.gov (NT 04952298).

### Statistics

The endpoints will be evaluated using descriptive statistics, and the key figures of the distributions will be presented in tables. Statistical analyses will be made as described in previous studies which established the concept of benchmarking for other surgical procedures (10, 11, 15). Hereby, the cohort is stratified into patients with a low-preoperative and high-preoperative risk profile according to comorbidities which are proven to negatively impact the postoperative course. Afterward, the benchmark cut-off for each outcome indicator is set at the 75th percentile of the median of all centers (10). Depending on the composition of the data, χ2, Mann-Whitney U, and *t*-tests, or Pearson or Spearman correlation coefficients, will be conducted. The relationships between multiple independent variables on the dependent variable(s) will be tested using multivariate regression analysis.

### Registry Reports

The results of the EASINESS registry will be published by the Steering Committee and distributed to all participating centers following careful analysis by the principal investigators (R.D. and T.S.) at the Department of Neurosurgery, University Medical Center Hamburg-Eppendorf.

## Discussion

In recent years, quality monitoring and improvement have become more important in the field of healthcare, especially in surgery (20, 21). As a various single- and multiple-outcome indicators for evaluating the quality of surgical procedures were introduced, the concept of benchmarking as introduced by Staiger et al. seems to be the most reliable concept for establishing standardized outcome references and defining acceptable outcomes (10). Benchmarking offers an opportunity to present valid, internationally applicable reference values which originate from the daily routine of a large representative cohort. This will be the first internationally validated benchmark data for neurosurgery and as such will demonstrate that such data collection is both feasible and useful.

Unlike data from randomized controlled trials, which have stringent patient selection criteria and questionable generalizability, this study has only a few patient-specific inclusion criteria, and the results will therefore be generalizable to usual neurosurgical practice.

The EASINESS is a retrospectively conducted, multicenter, open registry with narrowly defined inclusion criteria for international centers. The objective is the application of the previously described benchmarking to define robust and standardized outcome references after temporal lobe epilepsy surgery. Afterward, these benchmarks from an international cohort of high-volume centers will serve as reference values for the evaluation of novel surgical techniques and comparisons among centers or future clinical trials. Furthermore, EASINESS has been established with an interdisciplinary approach collecting outcome data from both a neurosurgical and epileptogenic perspective and presenting novel valuable data compared with existing registries.

### Limitations of This Study

The results of this registry should be interpreted considering the limitation that the study design is a retrospective, non-randomized design which could introduce some bias.

## Ethics Statement

The studies involving human participants were reviewed and approved by Local Ethics Committee Hamburg 2021-300051-WF. Written informed consent to participate in this study was provided by the participants' legal guardian/next of kin.

## Author Contributions

RD, LD, and TS contributed to conceptualization and design. All authors drafted the manuscript, critically revised it and collect the data.

## Conflict of Interest

The authors declare that the research was conducted in the absence of any commercial or financial relationships that could be construed as a potential conflict of interest.

## Publisher's Note

All claims expressed in this article are solely those of the authors and do not necessarily represent those of their affiliated organizations, or those of the publisher, the editors and the reviewers. Any product that may be evaluated in this article, or claim that may be made by its manufacturer, is not guaranteed or endorsed by the publisher.
